# Ubiquitin-specific protease 15 interacts directly with the HSV-1 alkaline nuclease and facilitates viral recombination and replication fork stability

**DOI:** 10.1128/jvi.00893-25

**Published:** 2025-08-18

**Authors:** Yee Vue, Patrick J. Mullon, Alexander Leehangin, Emiliano Maldonado-Luevano, Tyler D. Hasselmann, Kavi P. M. Mehta, Kareem N. Mohni

**Affiliations:** 1 Department of Molecular Medicine, Mayo Clinic 6915, Rochester, Minnesota, USA; 2 Department of Comparative Biosciences, University of Wisconsin 5228, Madison, Wisconsin, USA; University of Virginia, Charlottesville, Virginia, USA

**Keywords:** herpes simplex virus, DNA replication, DNA repair, iPOND, alkaline nuclease

## Abstract

Herpes simplex virus type 1 replicates in the nucleus of the host cell and utilizes many cellular proteins to facilitate DNA replication. HSV-1 DNA replication is closely linked with homologous recombination, and HSV-1 encodes a two-subunit recombinase consisting of an exonuclease (UL12) and a single-strand DNA-binding protein (ICP8). We identified the cellular deubiquitinating enzyme USP15 as a new UL12-binding partner. USP15 is one of the most enriched proteins on viral DNA and has recently been implicated in regulating cellular homologous recombination. Utilizing isolation of proteins on nascent DNA (iPOND) to compare the proteins on wild-type and UL12-deficient viruses, we observed that USP15 is not recruited to newly replicated DNA in the absence of UL12. We also observed that UL12 and USP15 interact directly *in vitro* using purified proteins. Together, these data suggest that UL12 directly recruits USP15 to viral DNA. UL12 stimulates a type of homology-directed repair called single-strand annealing (SSA). We generated a USP15 knockout cell line with an integrated GFP-based SSA reporter and observed that UL12 and USP15 both contribute to the maximal induction of SSA. In addition to the reporter cell line, there is a 10-fold reduction in the frequency of marker rescue, and individual replication forks move slower in the absence of USP15. These data support a role for USP15 in facilitating recombination during the virus life cycle and the maintenance of replication fork stability.

**IMPORTANCE:**

HSV-1 is a ubiquitous pathogen in the global population that causes lifelong latent infection with sporadic reactivation in the host. It has long been appreciated that HSV-1 DNA replication exhibits high rates of recombination and is linked to the host DNA damage response. In this study, we show a novel interaction between UL12, a member of the HSV recombinase, and the deubiquitinating host enzyme USP15. We show that USP15 physically interacts with UL12 and is required for UL12 to maximally stimulate recombination. In the absence of USP15, we also observe an overall reduction in virus growth. This work demonstrated that host proteins facilitate viral-induced recombination. The interaction of USP15 with UL12 represents a potential target for intervention against HSV-1 infection and associated disease.

## INTRODUCTION

Herpes simplex virus 1 (HSV-1) is a large double-stranded DNA virus that replicates in the nucleus of the host cell. The HSV genome consists of two unique regions (U_L_ and U_S_), which are flanked by inverted repeats. The U_L_ and U_S_ regions invert relative to one another, and there are high rates of recombination between coinfecting HSV viruses, and both activities require DNA replication (1–7). The replicating viral DNA also adopts a complex DNA structure that cannot enter a pulsed field gel even after digestion with a restriction enzyme that cuts once per unit length genome (8). In addition, analysis of the replicating DNA by electron microscopy reveals the presence of X- and Y-shaped structures indicative of recombination intermediates (9, 10). There is a large amount of evidence in support of recombination during the virus life cycle; however, little is known about the cellular proteins involved in this process.

HSV-1 encodes a two-subunit recombinase consisting of the 5′−3′ alkaline exonuclease, UL12, and the single-strand DNA-binding protein, ICP8, which also possesses strand annealing activity. Together, these two proteins can perform a strand exchange reaction *in vitro* and are reminiscent of the bacteriophage lambda Red α/β recombinase (11, 12). In addition, UL12 can stimulate double-strand break repair through the single-strand annealing (SSA) mechanism, a form of homology-directed repair characterized by extensive DNA end resection (13, 14). Together, these mechanisms are proposed to function in generating the longer-than-unit-length concatemers that are essential to packaging DNA (13, 15, 16). Indeed, the phenotype of the UL12-deficient virus, AN-1, exhibits near wild-type levels of viral DNA replication, but the DNA cannot be packaged into capsids, suggesting it is structurally aberrant (17–19).

HSV-1 DNA replication stimulates the cellular DNA damage response (DDR) kinase ATM (Ataxia-telangiectasia mutated) and results in phosphorylation of many ATM substrates involved in DNA double-strand break repair (20–22). In uninfected cells, double-strand breaks are sensed by the MRN (Mre11, Rad50, Nbs1) complex, which results in activation of ATM and phosphorylation of a large number of targets, including Nbs1, Chk2, Mdc1, and 53BP1 (23). In infected cells, ATM is activated early during infection, and the activation does not occur in the absence of DNA replication (20, 22, 24). Many DNA repair proteins, including ATM and the MRN complex, are recruited to the viral replication compartments and phosphorylated by ATM (20–22, 25–27). Utilizing a purification technique called isolation of proteins on nascent DNA (iPOND), ATM and Nbs1 can also be found specifically at HSV-1 DNA replication forks, suggesting a direct role in viral DNA replication (28–30).

Ubiquitin-specific protease 15 (USP15) is a newly identified ATM substrate and functions in homologous recombination. USP15 is recruited to double-strand breaks through Mdc1, is phosphorylated by ATM, and functions to regulate DNA end resection (31). In the absence of USP15, RPA and Rad51 are not recruited to sites of DNA damage, and BRCA1/BARD1 are not retained at sites of DNA damage. Furthermore, cells lacking USP15 are also defective in DNA repair through the homologous recombination pathway. The proposed mechanism of action is that USP15 deubiquitinates the BRCT domain of BARD1 to promote the retention of the BRCA1/BARD1 complex at sites of DNA damage (31).

USP15 is one of the most enriched human proteins on replicating viral DNA as measured by isolation of proteins on nascent DNA (iPOND) (28). In a search for new UL12-interacting proteins, we identified USP15 by mass spectrometry and demonstrated that the proteins directly interact *in vitro*. Utilizing iPOND with wild-type and UL12-deficient viruses, we show that USP15 recruitment to replicating DNA is completely dependent on the presence of UL12, suggesting that UL12 directly recruits USP15. We also demonstrate that viral replication is reduced in the absence of USP15. Specifically, we observe a reduction in the speed of viral DNA replication forks combined with a decrease in viral recombination as measured by SSA reporter assay and marker rescue. These data suggest that USP15 aids in viral replication by stimulating viral recombination and replication fork progression.

## RESULTS

### The HSV-1 alkaline nuclease, UL12, directly interacts with the cellular ubiquitin-specific protease 15

**Fig 1:**
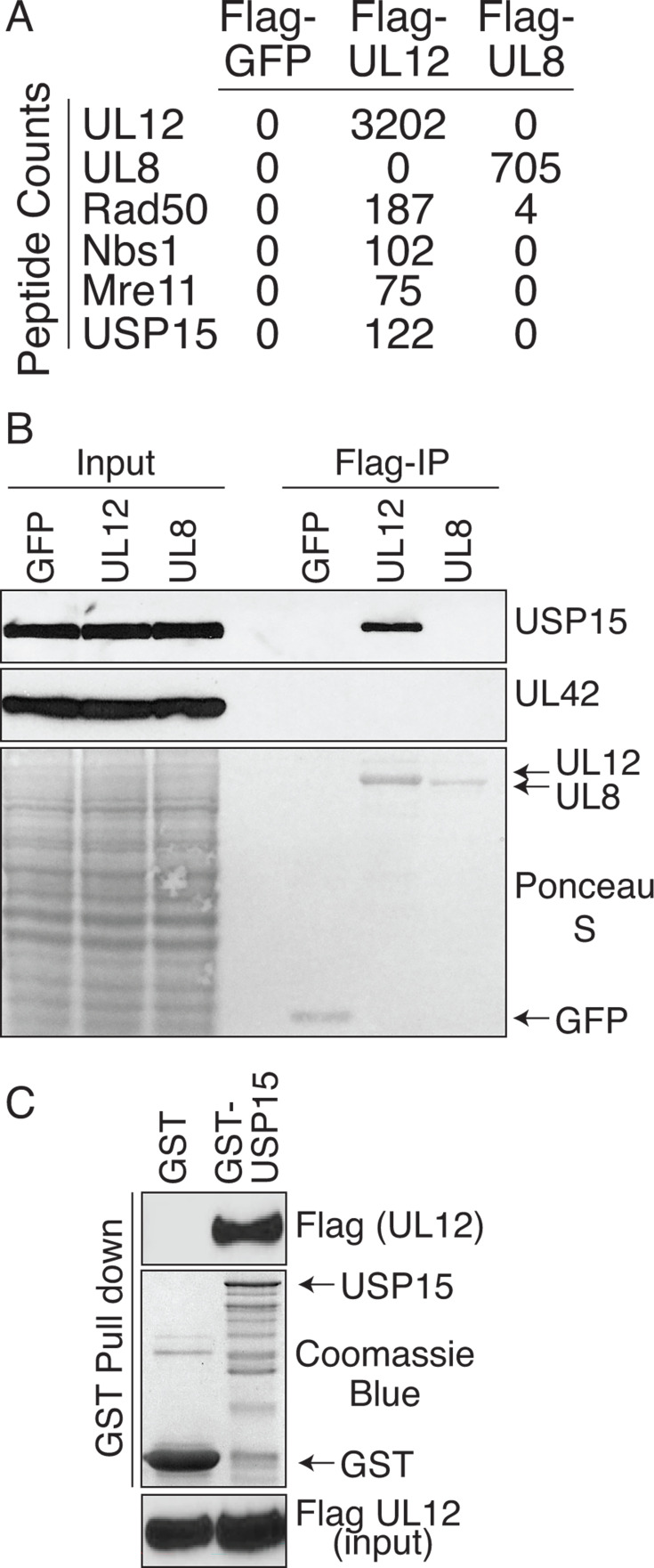
USP15 directly interacts with UL12. (**A**) Flag fusion proteins were immunoprecipitated, and interacting proteins were identified by mass spectrometry. The number of peptides identified for each protein is indicated. (**B**) Flag fusion protein immunoprecipitates immunoblotted with USP15 and UL42 antibodies. Ponceau S staining indicates the purified UL12, UL8, and GFP. (**C**) GST-USP15 was expressed in bacteria, and protein-bound beads were mixed with purified UL12. Elutions were immunoblotted with Flag antibody. Coomassie blue indicates the GST and GST-USP15. Mass spectrometry experiments were performed once, and all interacting proteins are indicated in Table S1. All other experiments in this figure were performed as three biological replicates, and a representative image is shown.

To better understand the role of UL12, we sought to identify UL12-interacting proteins. Flag-UL12 was expressed in 293T cells, and copurifying proteins were identified by mass spectrometry. We identified all three components of the MRN (Mre11, Rad50, and Nbs1) complex, a known interacting partner of UL12 (Fig. 1A) (15). In addition, we also identified similar numbers of peptides for USP15 to those associated with the MRN complex. Neither the MRN complex nor USP15 was identified in control purifications of Flag-GFP or Flag-UL8, suggesting that the interaction is specific to UL12 (Fig. 1A; Table S1). We further validated the mass spectrometry results by immunoblotting UL12 immunoprecipitations with an antibody raised against USP15. USP15 was only purified with UL12 and not the negative control proteins GFP or UL8 (Fig. 1B). Furthermore, we also show that none of the proteins interact with UL42, a component of the viral polymerase complex. To test whether the binding between UL12 and USP15 is direct, we purified UL12 from 293T cells to near homogeneity and combined it with bacterially expressed GST-USP15. UL12 only bound GST-USP15 and not GST alone, suggesting that the interaction is specific and does not require any additional proteins (Fig. 1C). The smaller bands seen in the GST-USP15 purification likely represent degradation products and are frequently observed in one-step purifications (31).

### UL12 recruits USP15 to sites of viral DNA replication

**Fig 2:**
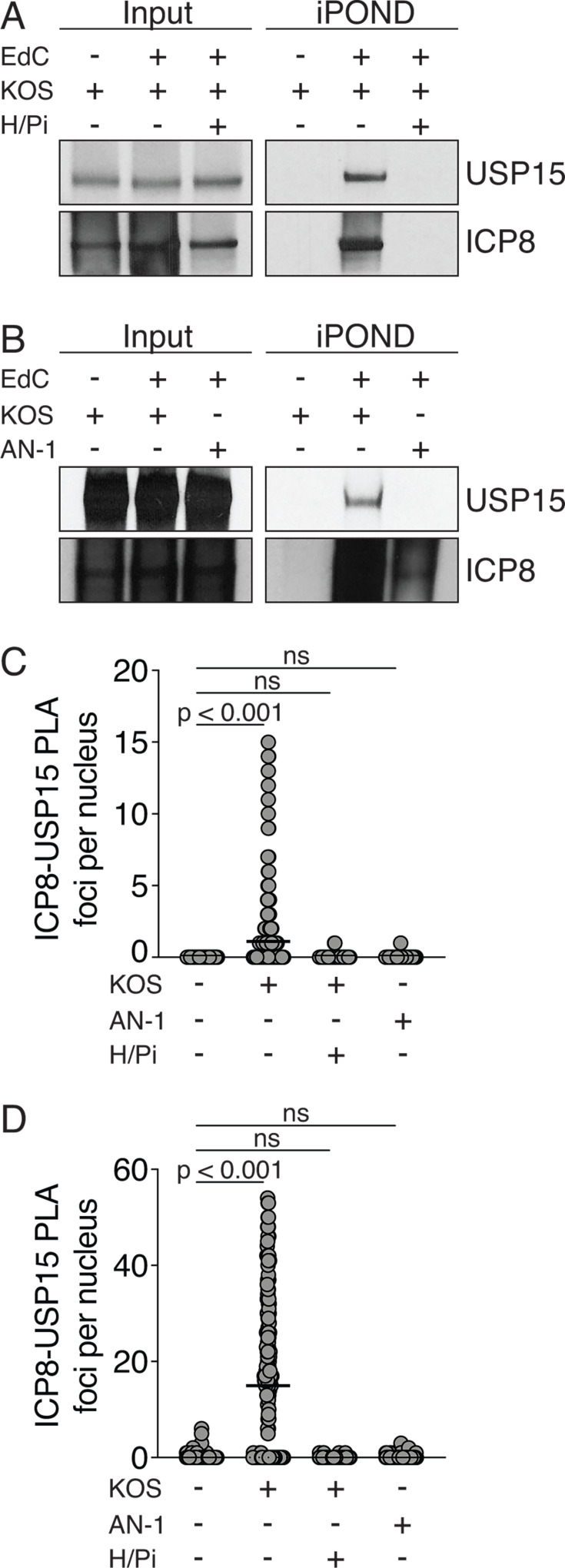
USP15 is recruited to replication forks by UL12. Cells were infected with KOS or AN-1 at an MOI of 2 PFU/cell and pulsed with 10 µM EdC at 6 hours post-infection and then processed for iPOND. (**A**) Immunoblotting of proteins associated with KOS forks compared to forks stalled with the viral helicase/primase inhibitor (H/Pi). (**B**) Immunoblot comparison of proteins at KOS versus AN-1 replication forks. All iPOND experiments were performed as three biological replicates, and a representative image is shown. (**C and D**) Proximity ligation assays with ICP8 and USP15 antibodies at an MOI of (**C**) 3 PFU/cell or (**D**) 10 PFU/cell. Each circle indicates the individual value per nucleus, and the line represents the median value. 100 nuclei were measured per condition in each biological replicate. Each PLA was performed twice, and one representative experiment is shown. One-way ANOVA with Tukey’s multiple comparisons post-test.

USP15 was recently reported to be one of the most enriched proteins at HSV-1 DNA replication forks using isolation of proteins on nascent DNA (iPOND) coupled with mass spectrometry (28). We confirmed this observation by infecting cells with KOS at a multiplicity of infection (MOI) of 2 PFU/cell in the presence or absence of the viral helicase/primase inhibitor, BAY 57-1293. Cells were pulsed with EdC for 10 minutes at 6 hours post-infection before being fixed and processed for iPOND. Immunoblots of the iPOND samples reveal a strong signal for USP15 and ICP8 in the KOS + EdC sample. Importantly, neither of these proteins were observed in the presence of the helicase/primase inhibitor or in the negative no-EdC control (Fig. 2A). These observations suggest that the observed USP15 recruitment is specific to newly replicated viral DNA. To test whether UL12 is required for USP15 recruitment, we infected cells with KOS or the UL12-deficient virus, AN-1, at an MOI of 2 PFU/cell. Cells were again pulsed with EdC for 10 minutes at 6 hours post-infection. Immunoblots of these iPOND samples demonstrate that USP15 is recruited to KOS + EdC samples but is not recruited to AN-1 + EdC (Fig. 2B). These data suggest that UL12 is required to recruit USP15 to viral DNA. We have previously reported that replication forks stall in the absence of UL12, and this could explain the slightly reduced capture of ICP8 in these experiments (32).

To further validate the interaction of USP15 with viral DNA, we performed proximity ligation assays (PLA) with antibodies for ICP8 and USP15. PLA uses secondary antibodies with complementary oligonucleotides. If the two secondary antibodies are close enough together, they can hybridize and act as primers for subsequent polymerase-based amplification with fluorescent nucleotides. The PLA signal is only generated when the two proteins are within 40 nm of each other, indicating a complex or close physical interaction. We observed positive PLA signal during infection with KOS at both low and high multiplicities of infection, and mock-infected cells served as a negative control. We did not observe any PLA signal in cells infected with KOS in the presence of the helicase/primase inhibitor or during infection with AN-1 (Fig. 2C and D). These observations correlate with the recruitment of USP15 to viral DNA via iPOND.

**Fig 3:**
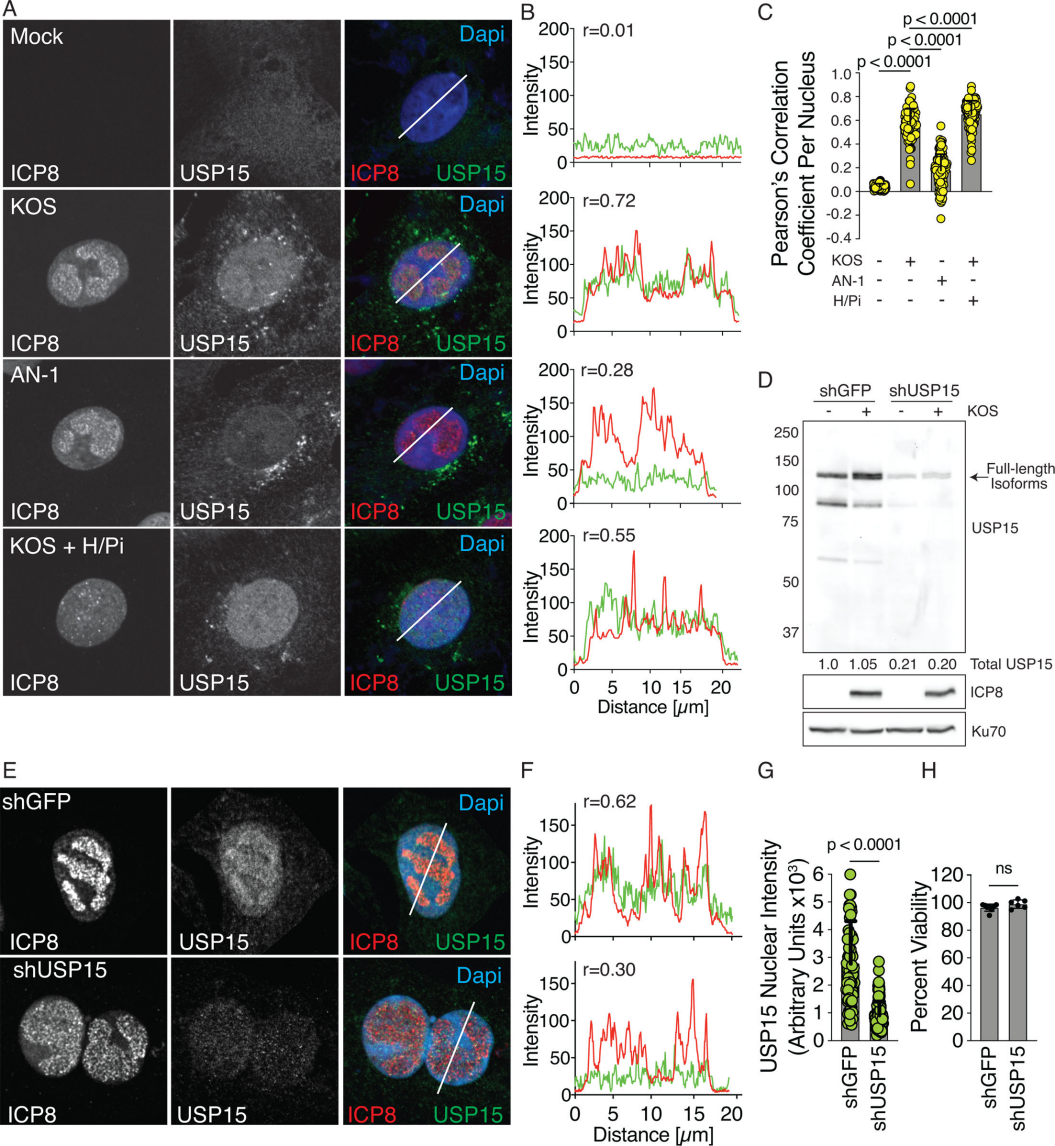
UL12 is necessary for the nuclear localization of USP15**.** Vero cells were infected with KOS or AN-1 at an MOI of 3 PFU/cell and fixed at 6 hours post-infection. Where indicated, helicase/primase inhibitor (H/Pi) was added at time 0. (**A**) Cells were stained with ICP8 and USP15 antibodies. All images were taken using the same laser intensities. (**B**) Traces were drawn and quantified using Zen software and correspond to the white line drawn on the merged image. Pearson correlation coefficients (R) between USP15 and ICP8 are indicated. 25 individual nuclei were analyzed with trace files per biological replicate. The experiment was performed twice, and one representative trace is shown. (**C**) Total nuclear USP15 colocalization with ICP8 per cell was quantified using CellProfiler. Mean ± SD, ANOVA with Dunnett’s multiple comparisons test, yellow circles indicate individual data points. 100 nuclei were measured per condition in each biological replicate. The experiment was performed twice, and one representative experiment is shown. (**D–H**) Vero cells were infected with lentiviruses expressing either shGFP or shUSP15. (**D**) Knockdown was determined by immunoblot analysis (**D**) and immunofluorescence analysis (**E**) of USP15. (**F**) Trace lines and Pearson correlation coefficients (R) between USP15 and ICP8 are indicated and analyzed as described above. (**G**) Total nuclear USP15 intensity per cell was quantified using CellProfiler. Mean ± SD, t test, green circles indicate individual data points. 100 nuclei were measured per condition in each biological replicate. The experiments in D-G were performed twice, and one representative experiment is shown. (**H**) Cell viability was determined with Alamar blue. Mean ± SD, t test, circles indicate technical replicates (*n* = 6). The experiment was performed twice, and one representative experiment is shown.

We also analyzed USP15 recruitment to sites of DNA synthesis using immunofluorescence. In uninfected cells, USP15 has a diffuse staining pattern throughout the nucleus and cytoplasm. Upon infection with KOS, we observed an enrichment of USP15 in the nucleus (Fig. 3A) similar to that of UL12 (15). Trace files of infected cells show colocalization of USP15 (green) and ICP8 (red) with a Pearson correlation coefficient of 0.72 (Fig. 3B). This nuclear localization was greatly reduced during infection with AN-1, although there is still detectable USP15 in the nucleus. Trace files of AN-1-infected cells show minimal colocalization of USP15 (green) and ICP8 (red) with a Pearson correlation coefficient of 0.28 (Fig. 3A and B). USP15 was also recruited to the nucleus during KOS infection in the presence of the viral helicase/primase inhibitor (Fig. 3A). To further validate these observations, we determined the Pearson’s correlation coefficient throughout the entire nucleus between USP15 and ICP8. We observed a strong colocalization during infection with KOS that was lost during infection with AN-1. Similarly, we also observed a correlation between USP15 and ICP8 during KOS infection in the presence of the viral helicase/primase inhibitor (Fig. 3C). These data suggest that UL12 expression is required for the colocalization of USP15 and ICP8, but not DNA replication.

To confirm that the increase in USP15 signal was specific to USP15, we performed several control experiments. First, Vero cells expressing shRNA targeting USP15 or a negative control protein, GFP, were infected with KOS at an MOI of 3 PFU/cell and analyzed by immunoblotting at 6 hours post-infection. The USP15 antibody recognized three main bands that were all reduced in the presence of shUSP15, consistent with the many reported alternatively spliced isoforms of USP15 (33). We did not observe any additional bands in infected samples, indicating the USP15 antibody is not cross-reacting with viral proteins (Fig. 3D). We also did not observe any significant change in the amount of total USP15 during infection with KOS. Second, we performed immunofluorescence for USP15 on shGFP and shUSP15. Trace files of shGFP cells show colocalization of USP15 (green) and ICP8 (red) with a Pearson correlation coefficient of 0.62, while trace files of shUSP15 cells showed a reduction in USP15 signal intensity and a reduced Pearson correlation coefficient of 0.30 (Fig. 3E and F). We then quantified the total USP15 intensity per nucleus and observed a significant decrease in USP15 intensity during infection of shUSP15 cells compared to shGFP cells (Fig. 3G). Finally, we confirmed that shUSP15 had no reduction in cell viability (Fig. 3H). Together, these data indicate the specificity of the USP15 antibody.

**Fig 4:**
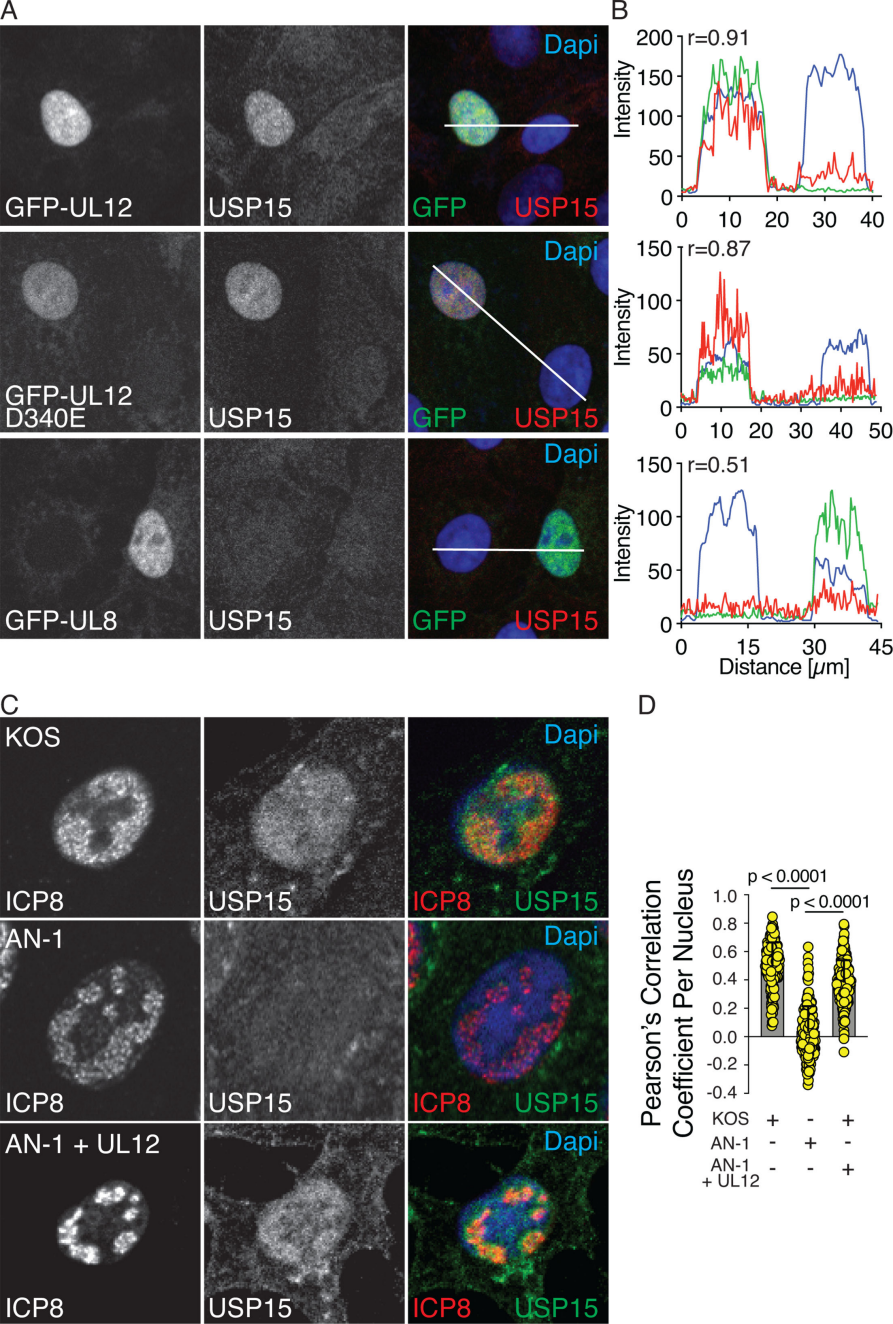
UL12 is sufficient for the nuclear localization of USP15. (**A and B**) Vero cells were transfected with plasmids expressing the indicated GFP fusion proteins and stained with USP15 antibody. Traces were drawn and quantified using Zen software and correspond to the white line drawn on the merged image. Pearson correlation coefficients (R) between USP15 and GFP are indicated. 25 individual nuclei were analyzed with trace files per biological replicate. The experiment was performed four times, and one representative trace is shown. (**C and D**) Vero cells were infected with KOS or AN-1 at an MOI of 10 PFU/cell and fixed at 6 hours post-infection. Where indicated, Vero cells were first transduced with lentivirus expressing UL12 prior to infection (+ UL12). Cells were stained with ICP8 and USP15 antibodies. All images were taken using the same laser intensities. Total nuclear USP15 colocalization with ICP8 per cell was quantified using CellProfiler. Mean ± SD, ANOVA with Dunnett’s multiple comparisons test, yellow circles indicate individual data points. 100 nuclei were measured per condition in each biological replicate. The experiment was performed twice, and one representative experiment is shown.

Next, we transiently transfected cells with plasmids expressing GFP fusions of UL12 or UL8. Expression of UL12 and catalytically dead UL12 (D340E) resulted in the nuclear localization of USP15, while expression of UL8, an unrelated protein, did not. Trace files of UL12 and D340E transfected cells show nuclear localization of USP15 (red) only in GFP (green) expressing cells with a Pearson correlation coefficient of 0.91 and 0.87, respectively. This nuclear localization was not observed in UL8-expressing cells (Fig. 4). These data suggest that UL12 is sufficient for the nuclear localization of USP15 and that the nuclease activity of UL12 is not required. To further validate the role of UL12 and control for any unforeseen mutations in AN-1, we transduced Vero cells with a lentivirus expressing UL12 under the weak HSV gD promoter and then infected them with AN-1 and monitored USP15 localization to the nucleus and colocalization with ICP8. AN-1-infected Vero cells showed little recruitment of USP15 to the nucleus and minimal colocalization with ICP8. Expression of UL12 with the lentivirus during AN-1 infection completely rescued USP15 recruitment and colocalization with ICP8 (Fig. 4C and D). This indicates that the observations made during infection with AN-1 are specific to UL12.

### USP15 is required for efficient HSV-1 DNA replication

**Fig 5:**
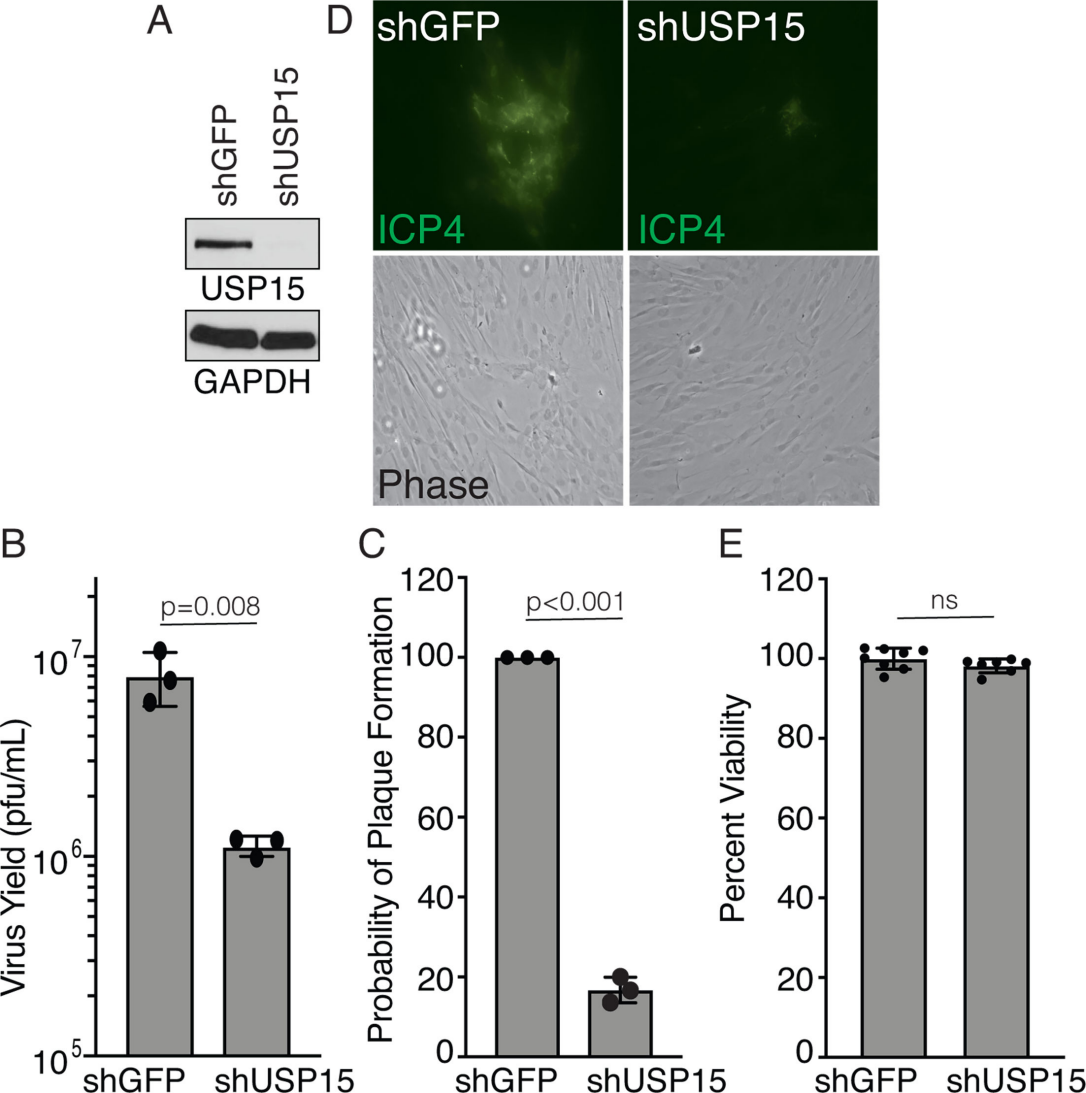
USP15 is required for efficient HSV replication. HFF cells were transduced with lentiviruses expressing either shGFP or shUSP15. (**A**) Knockdown was determined by immunoblot analysis of USP15. (**B**) Cells were infected with KOS at an MOI of 0.01 PFU/cell for 24 hours. Virus yield was determined by plaque assay on Vero cells. (**C**) Cells were infected with 200 PFU of KOS and overlayed with 2% human serum. Cells were fixed at 3 days post-infection and stained with crystal violet to visualize plaques. Experiments in A–C were performed as three biological replicates. One representative immunoblot is shown. Data from all biological replicates is reported in the graphs. (**D**) Cells were infected with KOS at an MOI of 0.005 PFU/cell and overlaid with 2% human serum. Cells were fixed at 24 hours post-infection, and plaques were visualized with immunofluorescence for ICP4. 25 individual plaques were analyzed per biological replicate. The experiment was performed twice, and one representative image is shown. (**E**) Cell viability was determined with Alamar blue, circles indicate technical replicates (*n* = 8). The experiment was performed twice, and one representative experiment is shown. All graphs show mean ± SD, t test.

To test whether USP15 is required for HSV-1 replication, we utilized lentiviral delivery of shRNA targeting USP15 or a control protein, GFP. HFF cells were transduced with lentiviruses expressing shRNA, selected with puromycin, and knockdown was verified by immunoblot analysis (Fig. 5A). To measure virus growth, knockdown cells were infected with KOS at an MOI of 0.01 PFU/cell. Knockdown of USP15 resulted in a 10-fold reduction in the amount of virus produced at 24 hours post-infection (Fig. 5B). To investigate this defect further, we determined the probability of plaque formation on USP15-depleted cells compared to the control cells. Confluent monolayers of cells expressing shGFP or shUSP15 were infected with 200 PFU KOS and overlayed with 2% human serum. Cells were fixed at 3 days post-infection and stained with crystal violet to visualize the plaques. USP15-depleted cells had a 5-fold to 10-fold reduction in the probability of plaque formation as compared to the control cells (Fig. 5C). Immunofluorescence analysis of ICP4 in plaque experiments revealed that plaques were much smaller in the absence of USP15 and infection did not spread to neighboring cells, likely explaining the reduced probability of plaque formation observed with crystal violet at 3 days post-infection. (Fig. 5D). Finally, we also confirmed that shUSP15 had no reduction on cell viability in HFF cells (Fig. 5E). Together, these data suggest that USP15 is required for efficient viral replication.

**Fig 6:**
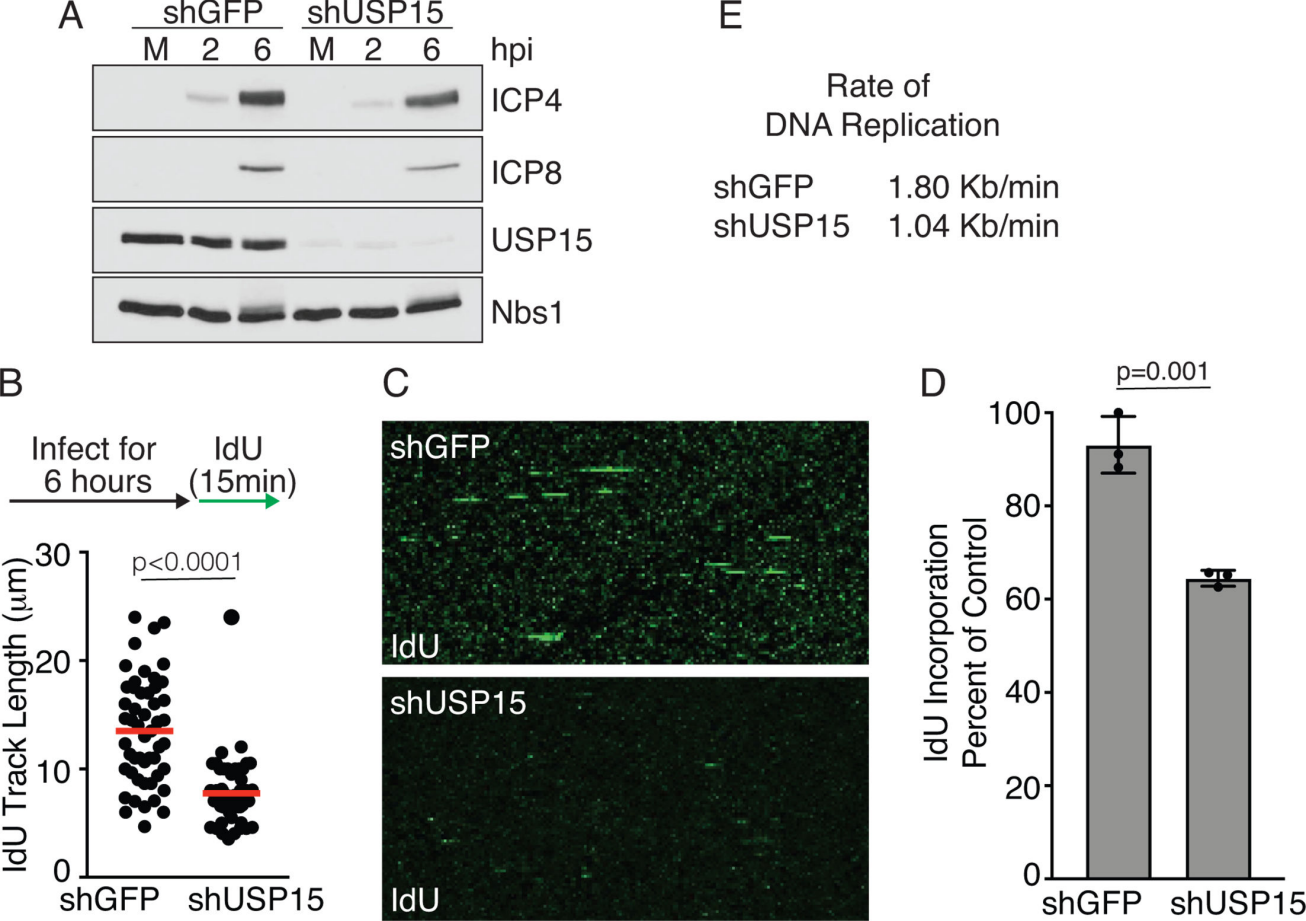
USP15 is required for HSV DNA replication fork progression. HFF cells were infected with lentiviruses expressing either shGFP or shUSP15 and then infected with KOS at an MOI of 2 (A) or 3 PFU/cell (B-E). (**A**) Immunoblot analysis of viral gene expression. (**B**) Single-molecule DNA combing of replication forks labeled with IdU for 15 minutes. The red line indicates mean, the t test, the circles indicate individual IdU fiber lengths. (**C**) Representative images of data shown in panel B. Experiments in A–C were performed as three biological replicates. One representative immunoblot is shown. 50–100 fibers were measured per condition in each biological replicate, and one representative experiment is shown. (**D**) Total viral and cellular DNA was isolated from samples processed in parallel to panel B. DNA was applied to a nylon membrane and slot blotted to quantify total IdU incorporation, mean ± SD, t test, circles indicate biological replicates (*n* = 3). (**E**) DNA replication fork rates derived from measurements in panel B.

The inability to form a plaque could be caused by defects in many stages of the virus’s life cycle. To look for changes in gene expression, we monitored the levels of representative Immediate Early and Early genes, ICP4 and ICP8. Cells were infected with KOS at an MOI of 2 PFU/cell, and protein levels of ICP4 and ICP8 were determined by immunoblot analysis. We did not detect significant changes in ICP4 or ICP8 protein levels in USP15-depleted cells compared to control cells (Fig. 6A). Since there were no apparent changes in gene expression/protein levels, we decided to look specifically at DNA replication. DNA combing is a technique to analyze replication fork progression at the single-molecule level. Cells are pulsed with the nucleoside analog IdU for 10–20 minutes to label replicating DNA, and the individual DNA molecules are separated by combing onto coverslips. The IdU can be stained with antibodies and visualized using immunofluorescence. Furthermore, because the DNA is combed at a constant rate, it is possible to directly compare the length of the combed molecules (34). Cells were infected with KOS at an MOI of 3 PFU/cell and pulsed with IdU for 15 minutes at 6 hours post-infection. The DNA was combed and analyzed by immunofluorescence, and the length of the individual DNA fibers was measured. Control cells had large readily visible fibers compared to shUSP15 cells (Fig. 6B and C). Since fibers isolated from shUSP15 cells were difficult to detect and were significantly shorter than control fibers, we wanted to determine whether there was a global reduction in DNA synthesis. To test this, we isolated total viral and cellular DNA from cells pulsed with IdU as described above. DNA was applied to a nylon membrane, and IdU incorporation was measured by quantitative immunoblotting with an antibody that detects IdU. We observed a significant reduction in IdU incorporation in shUSP15 cells compared to shGFP cells (Fig. 6D). Lastly, we determined a DNA replication fork rate of 1.80 kb/min for shGFP and 1.04 kb/min for shUSP15 (Fig. 6E). These data suggest that USP15 is required for HSV-1 DNA replication fork progression or stability. The total decrease in IdU incorporation could be due to the decrease in replication fork rate or a global decrease in the amount of DNA replication occurring.

### USP15 is phosphorylated during HSV-1 infection

**Fig 7:**
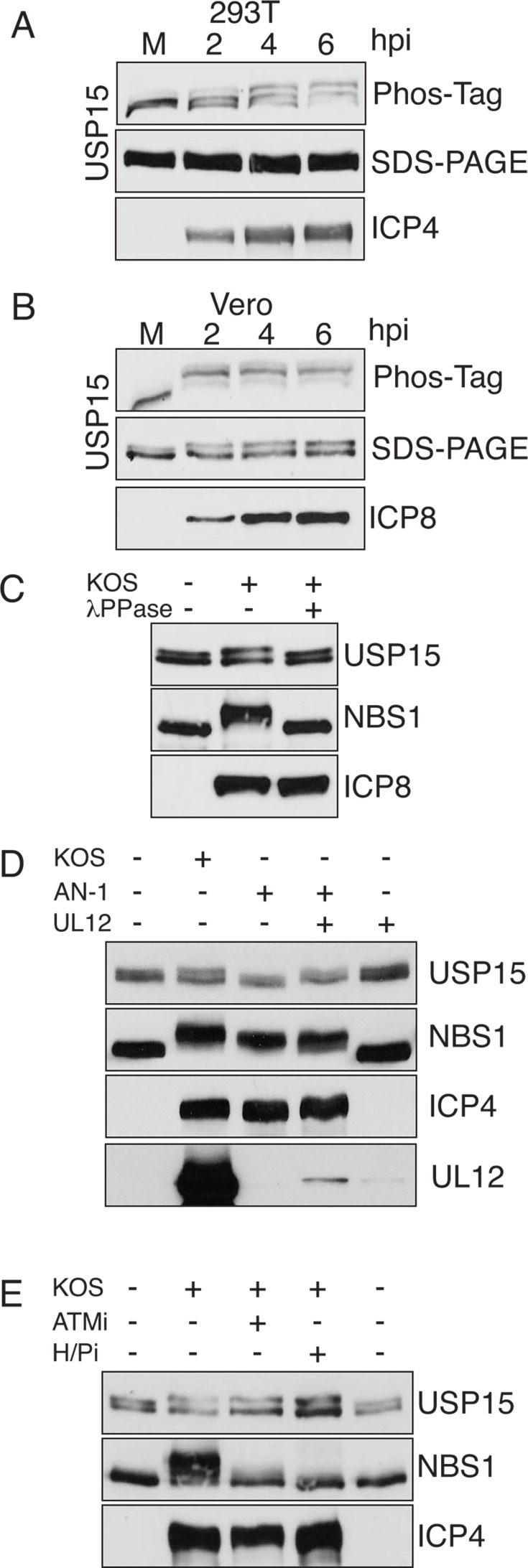
USP15 is phosphorylated during infection in a UL12-dependent manner. (**A**) 293T or (**B–E**) Vero cells were infected with KOS or AN-1 at an MOI of 10 and collected at 6 hours post-infection, unless indicated otherwise. (**A and B**) Cell lysates were separated by SDS-PAGE or with Phos-Tag acrylamide gels to visualize phosphorylated proteins and then immunoblotted with USP15, ICP4, and ICP8 antibodies. (**C**) Cell lysates were treated with lambda phosphatase (λPPase) for 30 minutes prior to separating proteins by SDS-PAGE and immunoblotting with USP15, Nbs1, and ICP8 antibodies. (**D**) Comparison of USP15 mobility by immunoblot during infection with KOS or AN-1. Where indicated, Vero cells were first transduced with lentivirus expressing UL12 prior to infection (+ UL12). (**E**) Infections were performed in the presence of a specific ATM inhibitor (ATMi) or viral helicase/primase inhibitor (H/Pi). All experiments were performed at least three times, and one representative immunoblot is shown in each panel.

USP15 is required for DNA double-strand break repair and functions downstream of the ATM kinase. USP15 is also a substrate of the ATM kinase after the induction of DNA damage (31). Since ATM is activated during KOS infection (20–22) and USP15 is required for efficient HSV DNA replication, we hypothesized that USP15 would also be phosphorylated. Vero and 293T cells were infected with KOS at an MOI of 10 PFU/cell and collected at different times post-infection. Cell lysates were resolved on low percentage SDS-PAGE gels to detect any mobility differences or resolved on gels supplemented with Phos-Tag acrylamide, which preferentially retards the mobility of phosphorylated proteins. Immunoblotting for USP15 showed that USP15 mobility was reduced on Phos-Tag gels in both cell lines, suggesting that the protein is phosphorylated (Fig. 7A and B). The phosphorylation occurred early and was readily visible at 2 hours post-infection. We also observed a small mobility shift of USP15 in the Vero cell lysates resolved by traditional SDS-PAGE. To confirm that this mobility shift was phosphorylation, we treated cell lysates with lambda phosphatase prior to resolving them on the gel. The upper USP15 band shifts up with infection and shifts back to mock levels with the addition of lambda phosphatase, further confirming that the mobility shift is due to phosphorylation (Fig. 7C). As an additional control, we monitored another ATM substrate that also binds UL12, Nbs1, that is known to be phosphorylated after infection (20–22). The Nbs1 band shifts after infection with KOS and is reduced to the uninfected size with the addition of lambda phosphatase.

Since UL12 is required for the nuclear localization of USP15 during infection, we hypothesized that UL12 might be required for USP15 phosphorylation. We infected Vero cells with KOS or AN-1 at an MOI of 10 PFU/cell and monitored the mobility shift in USP15 and Nbs1. The USP15 mobility shift did not occur during infection with AN-1, suggesting that UL12 is required for the phosphorylation (Fig. 7D). To further confirm the role of UL12, we infected Vero cells transduced with UL12 under the weak gD promoter with AN-1. Despite the lower expression due to the gD promoter, lentivirus-delivered UL12 was able to fully rescue the phosphorylation of USP15 during infection with AN-1. These data rule out any unforeseen mutations in AN-1. Interestingly, the other UL12-binding protein, Nbs1, was still phosphorylated during infection with AN-1, suggesting that these two phosphorylation events differ mechanistically. Activation of the ATM kinase and phosphorylation of Nbs1 require viral DNA replication (22). To test whether DNA replication was required for phosphorylation of USP15, we infected Vero cells with KOS in the presence of the viral helicase/primase inhibitor or a selective ATM inhibitor. We observed that USP15 was phosphorylated in the presence of either inhibitor (Fig. 7E). Nbs1 was not phosphorylated during infection with either inhibitor, confirming that both inhibitors are working. These data suggest that DNA replication is not required to phosphorylate USP15 and that there is an additional kinase involved.

### USP15 is required for maximal HSV-1 viral recombination

**Fig 8:**
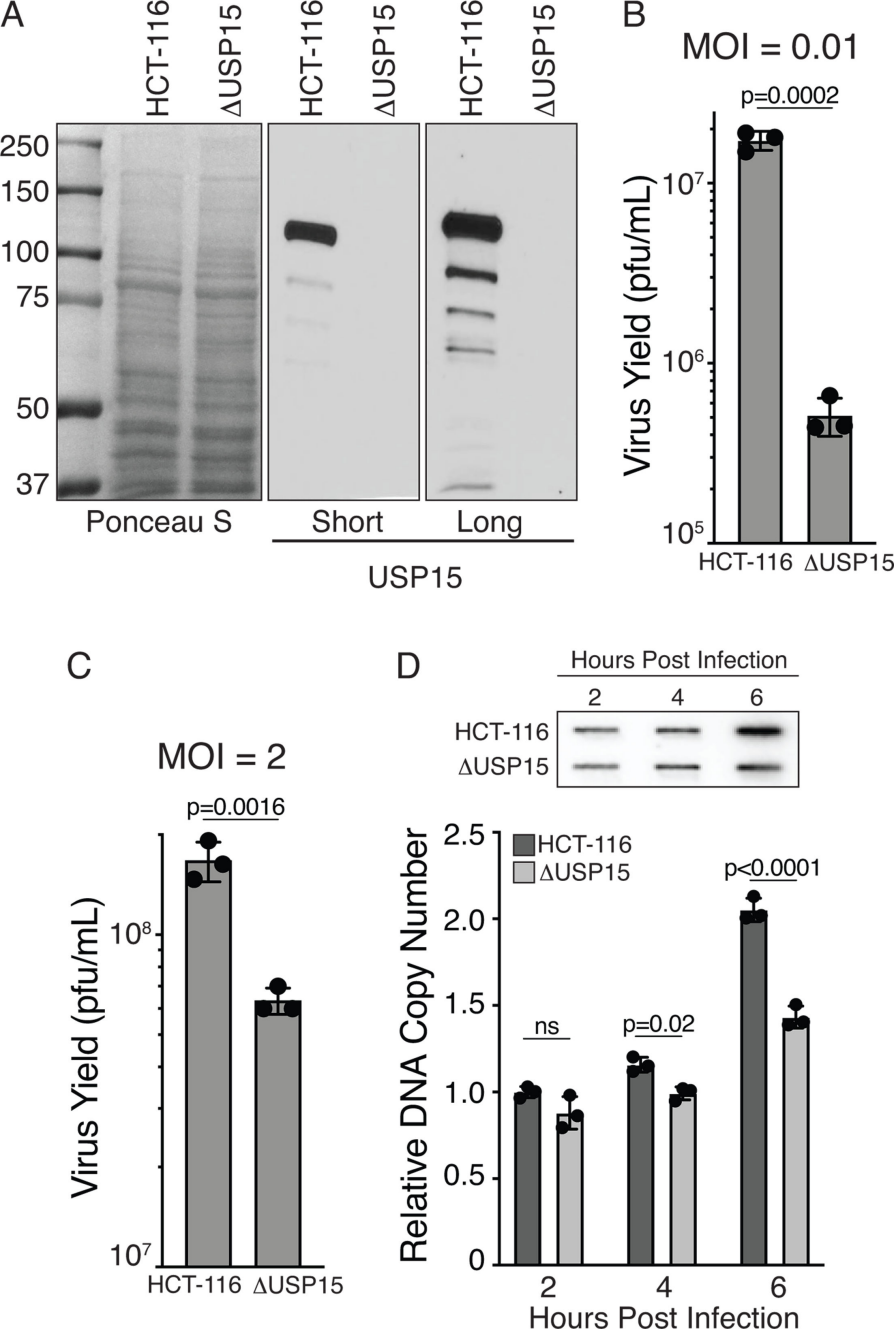
USP15 knockout cell line shows reduced virus yield and decreased levels of DNA replication. (**A**) Immunoblot of wild-type and ΔUSP15 HCT-116 cells. (**B and C**) Wild-type and ΔUSP15 cells were infected with KOS at an MOI of 0.01 or 2 PFU/cell for 24 hours. Virus yield was determined by plaque assay on Vero cells (*n* = 3). (**D**) Wild-type and ΔUSP15 cells were infected with KOS at an MOI of 2 PFU/cell, and total DNA was isolated at the indicated times post-infection. DNA was applied to a nylon membrane and Southern blotted with radio-labeled KOS genomic DNA to quantify total viral DNA. All graphs show mean ± SD, circles indicate biological replicates (*n* = 3). *P* values are from a t test (**B and C**) or an ANOVA (**D**).

We have previously published that UL12 can stimulate a specific type of DNA double-strand break repair called Single-Strand Annealing (SSA) (13). To test whether USP15 is required for SSA or for the ability of UL12 to stimulate SSA, we sought to generate a genetic system with knockout of USP15 followed by integration of a chromosomal SSA reporter assay as previously described for other genes involved in SSA (13, 14). We generated an HCT-116 USP15 knockout cell line using CRISPR/Cas9 and confirmed the loss of USP15 expression by immunoblot (Fig. 8A). Since all previous loss-of-function experiments were done in HFF cells, we wanted to confirm that the ΔUSP15 HCT-116 also had a reduced ability to support HSV-1 replication. We infected wild-type or ΔUSP15 cells with KOS at an MOI of 0.01 or 2 PFU/cell. Loss of USP15 resulted in a 10-fold reduction in the amount of virus produced at 24 hours, similar to what was observed with low MOI infections of HFF cells expressing shUSP15 (Fig. 8B and C). We also observed a more modest decrease of about 60% at the higher MOI of 2 PFU/cell. We also wanted to confirm the reduction in DNA synthesis observed in HFF shUSP15 cells. We infected wild-type or ΔUSP15 cells with KOS at an MOI of 2 PFU/cell and quantified total viral DNA by Southern blot at various times post-infection. The levels of viral DNA were equivalent at 2 hours post-infection, indicating equal amounts of input virus were added to each sample. By 6 hours post-infection, there was significantly more viral DNA in wild-type cells compared to ΔUSP15 cells (Fig. 6D). This is consistent with a reduced level of DNA replication in the absence of USP15.

**Fig 9:**
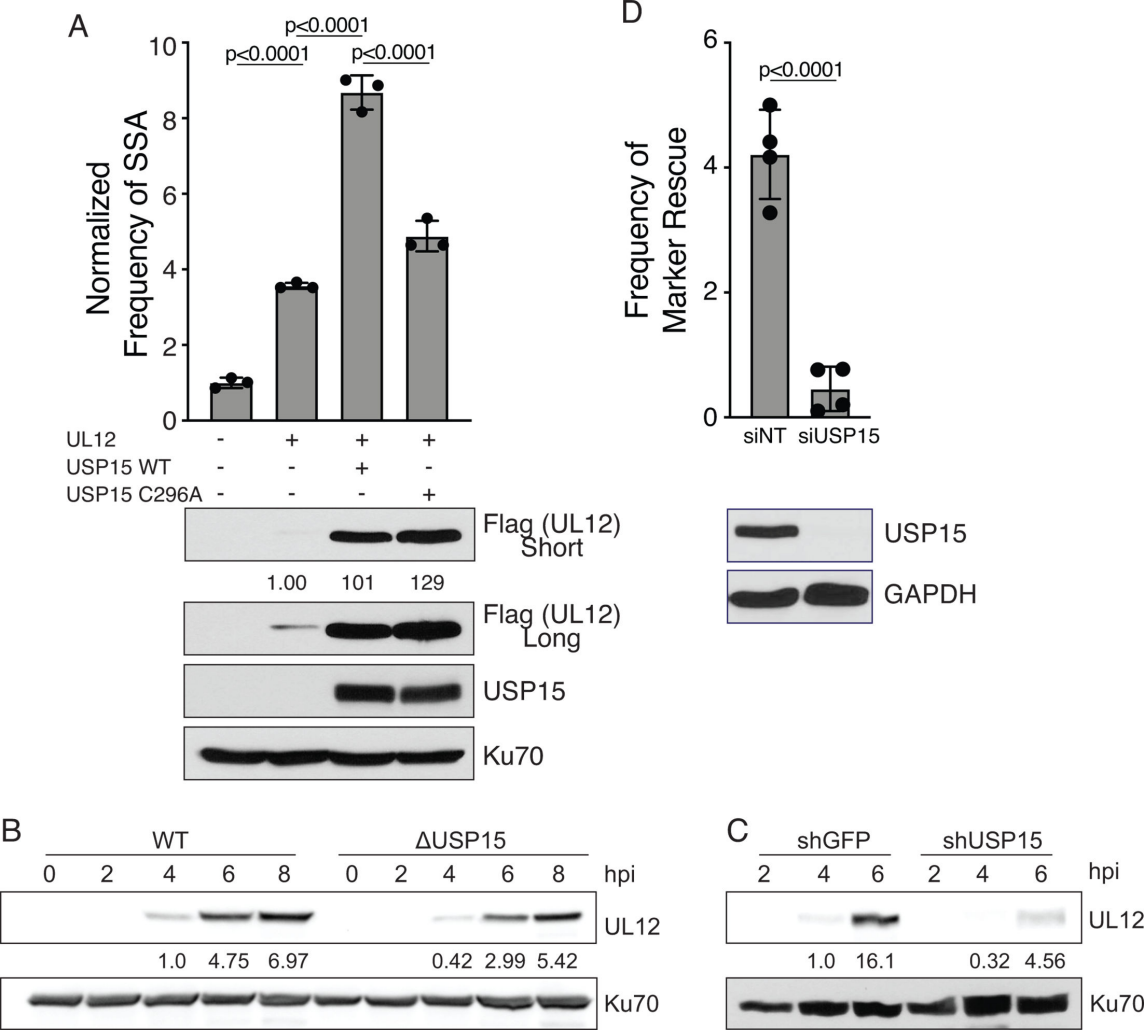
USP15 stabilizes UL12 and is required for maximal HSV viral recombination. (**A**) ΔUSP15 SA-GFP reporter cell line was transfected with an I-SceI expression vector and UL12, USP15, or empty vector as indicated. Samples were normalized to the I-SceI empty vector control without USP15 expression. The graph shows mean ± SD, ANOVA with Tukey’s multiple comparisons test, circles indicate biological replicates (*n* = 3). Representative immunoblot of UL12 and USP15 expression in transfected cells. Wild-type and ΔUSP15 HCT-116 cells (**B**) and HFF cells expressing either shGFP or shUSP15 (**C**) were infected with KOS at an MOI of 2 PFU/cell. Cells were collected at the indicated times post-infection and immunoblotted as indicated. Numbers represent the normalized levels of UL12. These experiments were done three times, and a representative immunoblot is shown. (**D**) U2OS cells were transfected with USP15 siRNA and then transfected 48 hours later with 100 PFU of infectious 0β DNA and a 10-fold molar excess of the wild-type correcting fragment from pW3. Samples were collected after maximal CPE was observed, and the frequency of blue and white plaques was determined on U2OS cells by staining with X-gal and neutral red. Graph shows mean ± SD, t test, circles indicate biological replicates (*n* = 4). Immunoblot of USP15 indicates the level of knockdown.

Next, we integrated the SSA reporter plasmid into the ΔUSP15 HCT-116 cells to generate ΔUSP15 SA-GFP cells. The SA-GFP reporter contains a split GFP gene with an I-SceI restriction site in the 3′ GFP sequence. Repair of the I-SceI-induced double-strand break by SSA restores GFP expression, which can be monitored by flow cytometry (14). ΔUSP15 SA-GFP cells were transfected with I-SceI and plasmids expressing UL12, USP15, or an empty vector control and analyzed for GFP expression 72 hours post-transfection. Cells transfected with I-SceI and empty vector were normalized to 1. UL12 was able to stimulate SSA eight- to nine-fold in the presence of USP15, similar to what we have previously reported in HCT-116 cells (13). In the absence of USP15, UL12 was only able to stimulate SSA three- to four-fold, suggesting that USP15 is not absolutely required for the induction of SSA by UL12. We also observed that UL12, in the presence of catalytically inactive USP15 C296A, was only able to stimulate SSA to the level of UL12 alone, suggesting both proteins are contributing to maximal levels of SSA (Fig. 9A).

We performed immunoblots of transfected cells to confirm expression of UL12 and USP15. We observed that UL12 levels were reduced in the absence of USP15 and could be rescued by co-expressing USP15 (Fig. 9A). This reduced level of UL12 could explain the reduced SSA observed in the absence of USP15. Surprisingly, catalytically dead USP15 C296A was able to completely rescue the levels of UL12 but was not able to rescue the full induction of SSA. These data suggest that UL12 levels alone do not correlate with maximal induction of SSA and that USP15 catalytic activity supports another step in SSA in addition to stabilization of UL12. We also observed reduced UL12 levels in KOS-infected ΔUSP15 HCT-116 and shUSP15 HFF cells, consistent with a role for USP15 in stabilizing UL12 (Fig. 9B and C).

Another measure of recombination during viral infection is marker rescue. Infectious DNA of a mutant virus is co-transfected with a wild-type correcting fragment of DNA, and the progeny viruses are screened for the frequency of wild-type clones. We isolated infectious DNA from the ICP0 mutant 0β, which has the ICP0 coding sequence replaced with the *lacZ* gene under the control of the ICP0 promoter. We co-transfected it with the PstI/SacI fragment from pW3, which contains the wild-type ICP0 genomic locus. U2OS cells, which complement the defect in 0β, were transfected with siRNA targeting USP15 or a non-targeting control sequence, and then transfected a second time with the infectious DNA and correcting fragment 48 hours later. Samples were harvested after maximal CPE was observed and titrated on U2OS cells. Plaques were stained with X-gal and neutral red, and the frequency of white plaques was determined. Marker rescue was reduced 10-fold in the absence of USP15 compared to the non-targeting control (Fig. 9D), suggesting that USP15 facilitates recombination.

## DISCUSSION

We identified the deubiquitinating enzyme, USP15, as a new binding partner of the viral alkaline nuclease, UL12. We were also interested in USP15 because it was identified as being highly enriched on replicating viral DNA in other proteomic studies (28). We demonstrated that UL12 interacts directly with USP15, and that UL12 is necessary and sufficient for USP15 localization to the nucleus and sites of DNA replication during infection. Depletion of USP15 with shRNA treatment caused a significant reduction in both virus yield, probability of plaque formation, and the rate of DNA synthesis, suggesting that USP15 activity promotes infection and DNA replication. In addition, we observed decreased rates of homology-directed repair using both the SSA reporter assay and marker rescue. USP15 also regulates the stability of UL12 in both transfection-based experiments and during infection. Finally, we observed that USP15 is phosphorylated early during infection, and the phosphorylation is independent of ATM or DNA synthesis.

USP15 is important for homologous recombination and is phosphorylated by ATM in response to DNA double-strand breaks (31). HSV-1 DNA replication activates the ATM kinase, so we hypothesized that USP15 would be phosphorylated during infection. Indeed, we observed phosphorylation of USP15 as early as 2 hours post-infection by mobility shift in SDS-PAGE. Surprisingly, neither an HSV DNA replication inhibitor nor a specific ATM inhibitor prevented the phosphorylation of USP15. Previous work used a phospho-specific antibody to observe ATM phosphorylation on USP15 S678 in response to double-strand breaks (31). Since we are observing phosphorylation as a mobility shift, we cannot formally exclude that this residue is not being phosphorylated. Recent phosphoproteomic analysis of HSV-1 infected cells identified five phosphorylated residues on USP15 clustered between amino acids 226–244 with S229 and T226 being the most abundant and increasing as much as four- to five-fold over mock-infected cells (35). Both of these sites are followed by a proline, which constitutes a cyclin-dependent kinase (CDK) consensus phosphorylation site. CDK inhibitors prevent the expression of immediate early and early genes during HSV-1 infection (36); therefore, it is not possible to see whether they prevent USP15 phosphorylation as UL12 expression is required. Other groups have also identified cell cycle-regulated phosphorylation of USP15 at S229 but have not identified the kinase responsible (37). We also cannot exclude the possibility that USP15 is phosphorylated by one of the HSV-1-encoded kinases.

USP15 also promotes the replication of human papilloma virus (HPV) and hepatitis B virus (HBV). HPV16 E6 protein stability is regulated by USP15. Overexpression of USP15 results in increased amounts of E6 and a longer half-life of the protein. Conversely, silencing of USP15 results in the destabilization of E6 (38). Similar observations were made with HBV HBx protein. Overexpression of USP15 extended the half-life of HBx, while siRNA depletion of USP15 reduced it (39). In both cases, the target protein interacts with USP15 by co-immunoprecipitation, and it is proposed that the protein stabilization is mediated by deubiquitination of the target protein. We demonstrate that USP15 directly interacts with UL12 and that in the absence of USP15, less UL12 is observed. Mechanistically, these observations could suggest an interaction comparable to USP15 and HPV16 E6 and HBV HBx. One model is that USP15 stabilizes UL12 during infection by removing ubiquitin signals that would lead to UL12 degradation through the proteasome. However, we observed that the catalytic activity of USP15 is not required for the stabilization of UL12, indicating that it is not directly removing ubiquitin from UL12. Our previous work has correlated SSA activity during HSV-1 infection with UL12 activity (13), and here we show that both UL12 and USP15 contribute to maximal SSA activity and contribute to the recombination activity seen during HSV-1 infection. We propose that UL12 binds USP15 and recruits it to the nucleus, where it becomes phosphorylated and then subsequently facilitates viral DNA replication and recombination. The catalytic activity of USP15 is not needed to stabilize UL12, but it is required for maximal levels of recombination. Future work will determine whether phosphorylation of USP15 is required to stabilize UL12 and promote recombination. This study, however, does not exclude the possibility that the UL12-USP15 interaction modulates other aspects of the DNA damage response to cause the increase in homology-directed repair.

## MATERIALS AND METHODS

### Cells and viruses

Vero, 293T, HCT-116, U2OS, and HFF-1 cells were obtained from the American Type Culture Collection. All cells except HFF-1 were maintained in Dulbecco’s modified minimal essential medium (DMEM) with 7.5% fetal bovine serum (FBS). HFF-1 cells were maintained in DMEM with 10% FBS. Vero cells complementing the loss of UL12, 6-5, were maintained in DMEM with 7.5% FBS and G418 to maintain selection for the UL12 cassette (18). The KOS strain was used as wild-type HSV-1. The UL12-deficient virus, AN-1, contains a 917 base pair deletion in the UL12 gene, which is replaced by *lacZ* under the control of the ICP6 promoter (17). The ICP0 deletion virus, 0β, has both copies of the ICP0-coding region replaced by the *lacZ* gene under the control of the ICP0 promoter (40).

HCT-116 ΔUSP15 cells were generated with CRISPR/Cas9. Cells were transfected with pSpCas9(BB)−2A-Puro 2 (Addgene plasmid number 48139 [41]) containing a guide RNA targeting the third exon of USP15 (5′-AAGGTGTTCCTTAAGTGACT) (pKM552) and selected with 2 µg/mL puromycin for 2 days prior to plating for individual clones. Clones were screened by immunoblotting for loss of USP15 expression with an antibody raised against the C-terminus of the protein. The ΔUSP15 cell line was also validated by sequencing the edited alleles.

### Plasmids

USP15 (p5953) and USP15 C269A (p5954) expressing pcDNA-Xpress-His-USP15 were a gift from Peter Howley (Addgene plasmids # 23216 and # 23217 [38]). USP15 was cloned into N-terminal tagged His-GST-3C (pBG101) to generate His-GST-3C-USP15 (pAL1) and pCMV-HA (Clontech) to generate pCMV-HA-USP15 (pAL103). UL12 from pSAK-UL12/12.5 (42) was cloned into pLEGFP-Flag-NLS (pDC1161) to generate pLEGFP-Flag-UL12 (pKM506), pLPG-Flag (pTB114) to generate pLPG-Flag-UL12 (pKM507), and pLPCX-Flag-HA (pKM331) to generate pLPCX-Flag-HA-UL12 (pAL38). UL12 D340E from pSAK-UL12/12.5 (42) was cloned into pDC1161 to generate pLEGFP-Flag-UL12 D340E (pKM537). UL8 from pCM-UL8 (43) was also cloned into pDC1161 and pTB114 to generate pLEGFP-Flag-NLS-UL8 (pKM508) and pLPG-Flag-UL8 (pKM509), respectively. The SA-GFP reporter plasmid and the I-SceI expression plasmid have been previously described (14, 44). Plasmid pW3 contains the entire genomic region for ICP0, including 5′ and 3′ sequences (45). Plasmid transfections were performed with polyethylenimine unless otherwise indicated. Plasmid pKM507 is a lentivirus-based vector and expresses UL12 under the weak HSV gD promoter. Where indicated, lentiviruses were packaged and transduced as previously described (46).

### Co-immunoprecipitation and mass spectrometry

Nuclear extracts were prepared by the method of Dignam from 293T cells transfected with Flag-tagged GFP, UL12, or UL8 and in the presence of 250U Pierce universal nuclease (47). Proteins were immunoprecipitated using EZview Red FLAG M2 affinity gel. Interacting proteins were eluted by the addition of FLAG peptide, TCA precipitated, and analyzed by two-dimensional liquid chromatography tandem mass spectrometry. Alternatively, immunoprecipitated proteins were eluted with FLAG peptides and analyzed by immunoblot.

### Protein purification

293T cells expressing Flag-UL12 were lysed for 1 hour with rotation at 4°C in lysis buffer (20 mM Tris pH 7.5, 150 mM NaCl, 1 mM MgCl_2_, 0.1% Triton X-100, 1 mM DTT, 1 mM NaF, 1 mM sodium orthovanadate, 5 µg/mL aprotinin, 5 µg/mL leupeptin, and 250U Pierce Universal Nuclease). UL12 was immunoprecipitated from clarified cell lysates with EZview Red FLAG M2 affinity gel overnight at 4°C. Beads were washed three times with lysis buffer and once with LiCl buffer (10 mM HEPES pH 7.9, 300 mM LiCl, 1.5 mM MgCl_2_, 20% glycerol, 0.1% Triton X-100, 1 mM DTT, 1 mM NaF, 1 mM sodium orthovanadate, 5 µg/mL aprotinin, and 5 µg/mL leupeptin), and twice with elution buffer (20 mM HEPES pH 7.9, 100 mM KCl, 1.5 mM MgCl_2_, 20% glycerol, 0.01% Nonidet P-40, 1 mM DTT, 1 mM NaF, 1 mM sodium orthovanadate, 5 µg/mL aprotinin, and 5 µg/mL leupeptin). Flag-UL12 was eluted with 300 µg/mL Flag peptide in elution buffer.

GST-USP15 was induced with 1 mM IPTG for 1 hour at 30°C in BL21 cells. Cell pellets were resuspended in NET buffer (25 mM Tris pH 8, 50 mM NaCl, 0.1 mM EDTA, 5% glycerol, 1 mM DTT, and 5 µg/mL aprotinin, and 5 µg/mL leupeptin), sonicated, and then Triton X-100 was added to a final concentration of 1%. Samples were incubated on ice for 30 minutes, and soluble lysates were combined with glutathione sepharose beads. Bound proteins were washed three times in NET buffer with 1% Triton X-100 and twice with UL12 elution buffer.

For interaction studies, GST-USP15 on glutathione sepharose beads was resuspended in UL12 elution buffer, mixed with purified UL12, and rotated for 1 hour at room temperature. An input sample was removed and then beads were pelleted and washed three times with UL12 elution buffer. Proteins remaining on the beads were released by boiling in 2× SDS sample buffer.

### Isolation of proteins on nascent DNA (iPOND)

iPOND was performed as described (48, 49). Briefly, infected cells were labeled with 10 µM EdC for 10 minutes. After labeling, cells were cross-linked in 1% formaldehyde/PBS for 10 min at room temperature, quenched using 1.25 M glycine, and washed three times in PBS. Collected cell pellets were frozen at −80°C, then resuspended in 0.25% Triton-X/PBS to permeabilize. Pellets were washed once with 0.5% BSA/PBS and once with PBS prior to the click reaction. The click reaction was completed in 1 hour, and the cells were lysed by sonication. Capture of DNA-protein complexes utilized streptavidin-coupled C1 magnabeads for 1 h. Beads were washed with lysis buffer (1% SDS in 50 mM Tris pH 8.0), low salt buffer (1% Triton X-100, 20 mM Tris pH 8.0, 2 mM EDTA, and 150 mM NaCl), high salt buffer (1% Triton X-100, 20 mM Tris pH 8.0, 2 mM EDTA, and 500 mM NaCl), lithium chloride wash buffer (100 mM Tris pH 8.0, 500 mM LiCl, and 1% Igepal), and twice in lysis buffer. Captured proteins were eluted and cross-links were reversed in SDS sample buffer by incubating for 30 min at 95°C. Where indicated, 100 µM BAY 57-1293 was added immediately after infection.

### Proximity ligation assay

Cells were fixed with 4% paraformaldehyde for 10 minutes at 6 hours post-infection, and then permeabilized in 1% Triton-X 100 in PBS for 10 minutes. Proximity ligation assays were performed using the Duolink PLA mouse/rabbit kit (Sigma) according to the manufacturer’s suggested protocol as previously described (50). PLA foci per nucleus were determined using CellProfiler (51).

### Immunofluorescence analysis

Immunofluorescence (IF) was performed as previously described (52). Briefly, cells on glass coverslips were washed with PBS, fixed for 10 minutes with 4% paraformaldehyde, and then permeabilized for 2 minutes with ice-cold acetone. Cells were blocked with 3% normal goat serum in PBS and then reacted with antibodies as indicated. Primary antibodies include rabbit monoclonal anti-USP15 D1K6S (1:100; Cell Signaling Technologies 66310S), mouse monoclonal anti-ICP8 11E2 (1:50; Santa Cruz sc-53330), and mouse monoclonal anti-ICP4 10F1 (1:50; Santa Cruz sc-56986). Alexa Fluor secondary antibodies (1:200, Invitrogen) were used with fluorophores excitable at wavelengths of 488 and 594. Where indicated, 100 µM BAY 57-1293 was added immediately after infection. Vero cells were transfected with Lipofectamine with PLUS reagent (Invitrogen) according to the manufacturer’s suggested protocol. Trace files were generated with Zen software, and data were analyzed and graphed using Prism 10. USP15 intensity or colocalization with ICP8 was determined using CellProfiler (51).

### Virus growth and probability of plaque formation

Vero or HFF-1 cells were transduced with pLKO.1-based lentiviruses (Addgene plasmid number 10878 [53]) expressing shGFP (5′-GCAAGCUGACCCUGAAGUUCA) or shUSP15 (5′-CUUUAACAGAAAUUGUCUC) and selected with puromycin. Viruses were packaged by co-transfection of 293T cells with psPAX2 (Addgene plasmid number 12260) and pMD2.G (Addgene plasmid number 12259) as described (52). At 72 hours post-transduction, cells were infected with KOS at an MOI of 0.01 PFU/cell. Samples were collected at 24 hours post-infection, and virus titers were determined by plaque assay on Vero cells. For the probability of plaque formation, the transduced cells were infected with 200 PFU of KOS, as determined by plaque assay on Vero cells, and overlayed with methylcellulose. Cells were fixed at 72 hours post-infection, and plaques were visualized by staining with crystal violet. Alamar blue was used to measure cell viability in 96-well dishes as previously described (54). Data are presented as the mean of 6–8 technical replicates.

### Molecular combing and nucleoside incorporation

Cells were infected with KOS at an MOI of 3 PFU/cell. At 6 hours post-infection, cells were pulsed with 10 µM IdU for 15 minutes. For molecular combing, DNA was extracted using the Fiber Prep DNA Extraction Kit (Genomic Vision) and combed onto silanized coverslips using a Genomic Vision combing apparatus. Samples were then stained with mouse monoclonal anti-BrdU B44 clone (1:100; BD Biosciences 347580) that cross-reacts with IdU. Imaging and fiber measurements were performed as previously described (55).

To measure total nucleoside incorporation, DNA was isolated using the Wizard® Genomic DNA purification Kit (Promega) according to the manufacturer’s suggested protocol. The DNA was diluted in 6× SSC and boiled for 10 min at 100°C, cooled on ice, and then applied to a nylon membrane via a slot blot apparatus. The membrane was then denatured for 10 min on Whatman paper soaked in denaturation solution (1.5M NaCl, 0.5M NaOH). Then the membrane was neutralized for 5 min on Whatman paper soaked in neutralization solution (1M NaCl, 0.5M Tris pH 7.5). The membrane was then air-dried, and DNA was cross-linked with 1,200 J/m^2^ of UVC radiation. The membrane was blocked with 5% BSA in 1× TBST for 30 minutes at room temperature and probed with mouse anti-BrdU antibody B44 (BD Bioscience) diluted 1:1,000 in 0.1% BSA. The membrane was imaged on an iBright system for chemiluminescent signal, and the relative intensity of the signal was quantified using Invitrogen’s iBright analysis software.

### Southern blotting

DNA was isolated from infected cells and applied to a nylon membrane with a slot blot apparatus as described above. After crosslinking, the membrane was probed with radio-labeled KOS genomic DNA labeled with Prime-a-gene random primer labeling system (Promega). Probing and detection were performed as previously described (50).

### Single-strand annealing assay

HCT-116 USP15Δ SA-GFP reporter cell line was generated by transfecting the cells with the SA-GFP reporter plasmid and selecting for stable integration with Puromycin as previously described (13). Individual clones were screened for a functional reporter by transfecting an empty vector or the I-SceI and USP15 expression plasmid. Cells were analyzed 72 hours post-transfection by flow cytometry for GFP expression. For experiments, cells were transfected using FuGene HD and plasmids I-SceI, UL12, and USP15 as indicated and analyzed at 72 hours post-transfection. The empty vector plasmid was used to normalize DNA concentrations in all conditions.

### Marker rescue

Marker rescue was performed as previously described (56, 57). U2OS cells were transfected with 10 nM Qiagen All Star Negative control siRNA or Dharmacon ON-TARGET plus USP15 siRNA using Dharmafect 1 (Invitrogen) (54). After 48 hours, cells were transfected with 100 PFU of 0β infectious DNA and a 10-fold molar excess of the PstI/SacI fragment from pW3 with Lipofectamine PLUS. After maximal cytopathic effect was observed, samples were scraped into the media, freeze-thawed, and frozen at −80°C. Samples were titrated on U2OS cells, and when plaques were visible, they were stained with X-gal and neutral red. 0β expresses the *lacZ* gene under the control of the ICP0 promoter and forms blue plaques when stained with X-gal. Successful recombination with the wild-type PstI/SacI correcting fragment results in loss of *lacZ* and a white plaque phenotype. Frequency is determined by [(number of white plaques)/(total number of plaques)] × 100.

### Cell lysates and lambda phosphatase treatment

Whole-cell lysates were prepared with lysis buffer (50 mM Tris pH 8, 150 mM NaCl, 1 mM MgCl_2_, 0.1% Triton X-100, 1 mM DTT, 1 mM NaF, 1 mM sodium orthovanadate, 5 µg/mL aprotinin, 5 µg/mL leupeptin, and 250 U/mL Pierce Universal Nuclease) on ice for 30 minutes. Where indicated, 24 µL of clarified lysate was combined with 3 µL of 10× NEBuffer for MetalloPhosphatases (PMP), 2 µL 10X NEB MnCl_2_, and 400U Lambda Protein Phosphatase (New England Biolabs). No phosphatase inhibitors were included in the lysis buffer. Samples were incubated for 30 minutes at 30°C and then 2× SDS sample buffer was added in a 1:1 ratio prior to boiling for 3 min at 95°C. Where indicated, 10 µM ATM inhibitor KU-55933 (Selleck Chemicals) or 100 µM BAY 57-1293 was added immediately after infection.

### Immunoblotting

Clarified lysates were mixed with 2× SDS sample buffer and boiled before being resolved by SDS-PAGE and transferred to Nitrocellulose membranes. Where indicated, gels were supplemented with 50 µM Phos-Tag reagent (APExBIO) and 100 µM MnCl_2_ to retard the mobility of phosphorylated proteins. Membranes were blocked for 1 hour with 5% non-fat dry milk dissolved in TBST. Primary antibodies were diluted in bHead2 locking solution and incubated overnight at 4°C. Primary antibodies include mouse monoclonal anti-FLAG M2 (1:10,000; Sigma F1804), rabbit monoclonal anti-USP15 D1K6S (1:10,000; Cell Signaling Technologies 66310S), rabbit polyclonal anti-ICP8-367 (1:10,000; provided by William Ruyechan, State University of New York, Buffalo, NY) (58), rabbit polyclonal anti-UL12 BWpUL12 (1:10,000; provided by Joel Bronstein and Peter Weber), mouse monoclonal anti-ICP8 11E2 (1:1,000; Santa Cruz sc-53330), mouse monoclonal anti-GAPDH 6C5 (1:10,000; MilliporeSigma MAB374), mouse monoclonal anti-ICP4 10F1 (1:1,000; Santa Cruz sc-56986), rabbit polyclonal anti-Nbs1 (1:10,000; Bethyl A301-289A), rat monoclonal anti-HA 3F10 (1:10,000; MilliporeSigma 11867423001), and rabbit monoclonal anti-Ku70 D10A7 (1:5,000; Cell Signaling Technologies 4588).

### Quantification and statistical analysis

Statistical analyses were completed using Prism. An ANOVA test was used when comparing more than two groups, followed by a Dunnett’s or Tukey’s multiple comparisons post-test. A two-tailed t test was used to compare two samples with normally distributed data. No statistical methods or criteria were used to estimate sample size or to include/exclude samples. A combination of shRNA and CRISPR/Cas9-derived knockout cell lines was analyzed to confirm results were not caused by off-target effects or clonal variation. Experiments performed with AN-1 were validated with lentiviral expression of UL12 prior to infection with AN-1 to confirm that UL12 could rescue the observed phenotypes. Expression of UL12 alone was also used to confirm that the results were due to loss of UL12. Unless otherwise stated, all experiments were performed twice, and representative experiments are shown. For immunofluorescence and PLA experiments, at least 100 nuclei were quantified for each condition in every experiment.

## Data Availability

All data are present in the paper or supplemental table.

## SUPPLEMENTAL MATERIAL

Table S1 legend jvi.00893-25-s0001.docx Legend for Table S1.

Table S1 jvi.00893-25-s0002.xlsx Mass spec data.

ASM does not own the copyrights to Supplemental Material that may be linked to, or accessed through, an article. The authors have granted ASM a non-exclusive, world-wide license to publish the Supplemental Material files. Please contact the corresponding author directly for reuse.
