# Supplementary material for: Ubiquitin-specific protease 15 interacts directly with the HSV-1 alkaline nuclease and facilitates viral recombination and replication fork stability
Source: J Virol. 2025 Aug 18;99(9):e00893-25. doi: 10.1128/jvi.00893-25 (PMC12455970; doi:10.1128/jvi.00893-25)
Supplement: Table S1 legend — Legend for Table S1. [file jvi.00893-25-s0001.docx]

Supplemental Material Figure Legends:

Table S1: Spectral counts of all identified interacting proteins by IP-MS.
